# Chemical Composition, Enantiomeric Distribution and Antimicrobial, Antioxidant and Antienzymatic Activities of Essential Oil from Leaves of *Citrus* x *limonia*

**DOI:** 10.3390/molecules30040937

**Published:** 2025-02-18

**Authors:** Eduardo Valarezo, Laura Toledo-Ruiz, Wolter Coque-Saetama, Alfredo Caraguay-Martínez, Ximena Jaramillo-Fierro, Nixon Cumbicus, Miguel Angel Meneses

**Affiliations:** 1 Departamento de Química, Universidad Técnica Particular de Loja, Loja 1101608, Ecuador; afcaraguay@utpl.edu.ec (A.C.-M.); xvjaramillo@utpl.edu.ec (X.J.-F.); mameneses@utpl.edu.ec (M.A.M.); 2 Carrera de Ingeniería Química, Universidad Técnica Particular de Loja, Loja 1101608, Ecuador; lvtoledo@utpl.edu.ec; 3 Carrera de Bioquímica y Farmacia, Universidad Técnica Particular de Loja, Loja 1101608, Ecuador; wacoque@utpl.edu.ec; 4 Departamento de Ciencias Biológicas y Agropecuarias, Universidad Técnica Particular de Loja, Loja 1101608, Ecuador; nlcumbicus@utpl.edu.ec

**Keywords:** biological activity, GC-MS, (−)-(4*S*), (+)-(4*R*), limonene, (+)-(1*R*,5*R*)-*β*-pinene, (−)-(1*S*,5*S*)-*β*-pinene

## Abstract

*Citrus* x *limonia* is an aromatic species belonging to the Rutaceae family. In the present study, the chemical composition, enantiomeric distribution, and biological activity of the essential oil isolated from leaves of *Citrus* x *limonia* were determined. The essential oil was extracted through hydrodistillation. The chemical composition of the essential oil was determined by gas chromatography (GC) coupled to a flame ionization detector (GC-FID), and a mass spectrometer detector (GC-MS) using a nonpolar column. The enantiomeric distribution was performed using two enantioselective chromatographic columns. Antimicrobial activity was determined using the broth microdilution method. The antimicrobial activity was tested against eight bacteria and two fungi. The antioxidant activity was determined through ABTS and DPPH methods. The spectrophotometric method was used to determine anticholinesterase activity. In the essential oil, forty-three compounds were identified. These compounds represent 99.13% of the total composition. Monoterpene hydrocarbons were the most representative group in number of compounds (fourteen) and in terms of relative abundance (65.67%). The main constituent is found to be limonene (25.37 ± 0.80%), *β*-pinene (23.29 ± 0.15%) and sabinene (8.35 ± 0.10%). Six pairs of enantiomers were identified in the essential oil from fruits of *Citrus* x *limonia*. The essential oil showed moderate antibacterial activity against Gram-positive cocci *Enterococcus faecalis,* and Gram-positive bacillus *Lysteria monocytogenes* with a MIC of 1000 μg/mL. The oil exhibited strong antifungal activity against fungi *Aspergillus niger*, and yeasts *Candida albicans* with a MIC of 250 and 500 μg/mL, respectively. The antioxidant activity of essential oil was weak in ABTS method with a SC_50_ of 9.12 mg/mL. Additionally, the essential oil presented moderate anticholinesterase activity with an IC_50_ of 71.02 ± 1.02 µg/mL.

## 1. Introduction

Aromatic plants have been used since 5000 BC for their medicinal and aromatic properties. Aromatic plants are a rich source of fixed and volatile bioactive compounds [1]. Essential oils (EOs) are complex mixtures of volatile compounds obtained from various parts of plants, including leaves, flowers, stems, roots, bark, and fruits. These volatile secondary metabolites are characterized by their high volatility and strong aromas, performing ecological functions such as attracting pollinators and defending against pathogens and herbivores. The EOs are characterized by antimicrobial, antioxidant, and anti-inflammatory, among other pharmacological, properties, which makes them of interest for industries such as pharmaceutical, cosmetics and food industries. Their chemical composition varies according to the plant species and the extraction conditions, but generally, they include terpenoids (monoterpenes and sesquiterpenes), and phenolic compounds (phenylpropanoids), which are responsible for their biological, and organoleptic properties [2,3,4,5].

Among the botanical families known to produce essential oils, the Rutaceae family has a significant role. This family includes more than 158 genera and approximately 2013 species, predominantly distributed in tropical and subtropical regions around the world [6]. Within the Rutaceae, the *Citrus* genus is especially important, valued not only for its edible fruits, but also for essential oils with high medicinal and commercial value. The Rutaceae family, named after the *Ruta* genus, comprises plants characterized by their essential oil glands [7].

The *Citrus* genus falls within the subfamily Aurantioideae, a division within Rutaceae which is further split into two tribes: Clauseneae and Citreae. The Clauseneae tribe is regarded as more primitive, while the Citreae tribe, containing 28 genera, is divided into three subtribes: Triphasiinae, Balsamocitrinae, and Citrinae. The Citrinae subtribe, which includes the “true citrus” genera (*Fortunella*, *Eremocitrus*, *Poncirus*, *Clymenia*, *Microcitrus*, and *Citrus*), is distinguished by its unique fruit structure. The segmented fruits of Citrinae contain petiolate, fusiform pulp vesicles, forming a specialized structure known as hesperidia, characteristic of this subtribe, and absent in other Rutaceae plants [8].

Within the *Citrus* genus*,* the species *Citrus* x *limonia* (L.) Osbeck [9,10] is an introduced and cultivated plant, which is found across various regions of Ecuador. It is commonly found in Galapagos, the Coast, and the Andean region, especially in the provinces of Loja, Esmeraldas, Galapagos, Imbabura, Los Ríos, Pichincha, and Zamora Chinchipe [11]. The species *Citrus* x *limonia* is characterized by thorny, semi-deciduous trees, which have an open and irregular crown, along with a tortuous stem, and grayish bark, 3 to 6 m tall. Its leaves are simple, alternate, aromatic, with glands measuring 6 to 10 cm in length, with a non-winged petiole. It has large, thin, fragrant flowers, gathered in axillary summits. Its fruits are of the berry type, ellipsoid in shape, generally a small apical nipple, with a slightly rough surface, yellow-green color, and few seeds [12]. It thrives at elevations of 0–3000 m [13]. This species is valued for its high acidity, and distinct orange-colored peel and pulp [14].

The species *Citrus* x *limonia* is a hybrid of mandarin (*Citrus reticulata*), citron (*Citrus medica*), and a small genetic contribution from *Citrus micrantha*. In Ecuador, this species is known by the common names of *limón mandarina* or *limón chino*; however, worldwide, it receives common names such as *limón paraguayo* (Spanish), *limón misionero*, *lima mandarina*, *limón mandarino*, *limão capeta* (Portuguese), *laranja capeta*, *mandarin lime*, *lemandarin*, *rangpur lime* (English), *sylhet lime* or *surkh nimboo* (Hindi). Some of these common names are also used to refer to the species *Citrus limonia* Osbeck, which is possibly a hybrid between *Citrus reticulata* and *Citrus aurantifolia*, *Citrus limon* or *Citrus macrophylla* [8,15,16,17].

The *Citrus* x *limonia* fruit has a very acidic taste and features an orange-colored peel and pulp. Despite its name being frequently associated with limes, *Citrus* x *limonia* is not a true lime, lemon or mandarin, although it is often used as a substitute for limes in culinary contexts due to its high acidity. Regarding the botanical and phytochemical characteristics of *Citrus* x *limonia*, this species is a drought-resistant tree, and can grow up to 5–6 m tall, with aromatic green leaves and small, fragrant flowers. The fruit is known for its thin, shiny skin that turns from green to orange red. Its popularity extends beyond culinary uses, as it is also used for ornamental purposes, as well as a rootstock for grafting other citrus varieties [18].

The essential oils isolated from citrus have been studied abundantly . However, there are no studies on the chemical composition of the EO extracted from the leaves of the *Citrus* x *limonia* species. This fact has motivated the realization of this study with the aim of isolating and characterizing the physical properties, as well as the chemical profile, of the essential oil from leaves of *Citrus* x *limonia*. Furthermore, this research will examine the biological activity of itd essential oil focusing on antimicrobial, antifungal, antioxidant, and anticholinesterase activities.

## 2. Results

### 2.1. Essential Oil Isolated

A total of 4800 g of leaves from *Citrus* x *limonia* with a moisture level of 47.32 ± 3.56% *w*/*w* (fresh) were hydrodistilled in three different batches for the purpose of isolating the EO. From this plant material, 16.8 mL of EO were obtained, which represents a yield of 0.35 ± 0.02% (*v*/*w*) or 3.5 ± 0.2 mL/Kg.

### 2.2. Physical Properties of Essential Oil

The essential oil from leaves of *Citrus* x *limonia* was presented as an unctuous liquid of subjective yellowish green color, which was less dense than water. Table 1 shows the mean values of the physical properties of EO; additionally, the standard deviation (SD) is shown.

### 2.3. Chemical Composition of Essential Oil

The identification of EO compounds from leaves of *Citrus* x *limonia* was performed by gas chromatography coupled to mass spectrometry (GC-MS) while the abundance of the compounds was quantified by gas chromatography equipped with a flame ionization detector (GC-FID). Table 2 shows the compound number (CN), retention indices calculated (RIC), retention indices obtained from literature (RIR) [19,20], relative abundance (%) with its standard deviation (SD), group to which each compound belongs (GC), chemical formula (CF) and monoisotopic mass (MM).

**Figure 1:**
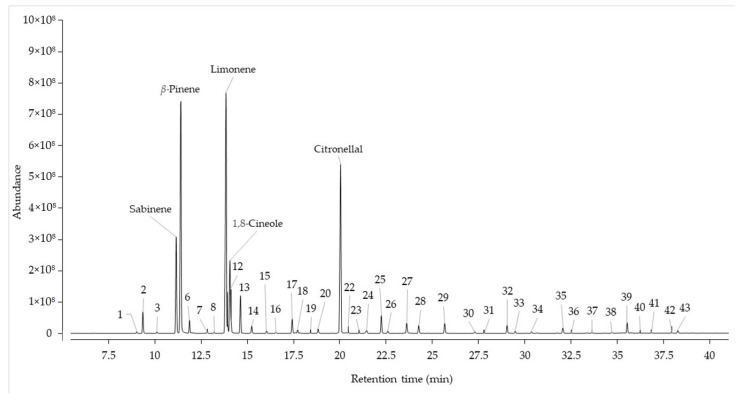
Chromatogram of the essential oil from leaves of *Citrus* x *limonia*. The peak numbers correspond to the compound numbers in Table 2.

Forty-three compounds were identified in the EO of *Citrus* x *limonia*, which represent 99.13% of the total composition. The compounds were grouped into five groups, according to the number of carbons (monoterpenes: 10 carbons, and sesquiterpenes: 15 carbons), the presence of oxygen (oxygenated and non-oxygenated), and one group of other compounds (non-terpenic compounds). Monoterpene hydrocarbon (MH) was the most representative group in number of compounds (fourteen), and in terms of relative abundance (65.67%). In fact, three of the five main compounds belong to this group. No compounds belonging to the diterpene (oxygenated and non-oxygenated) group were identified. Figure 1 shows the chromatogram of the EO from leaves of *Citrus* x *limonia*. The main constituents (>5%) (Figure 1), in order of abundance, are found to be MH (CF: C_10_H_16_, MM: 136.13 Da), limonene (mixture of (+) and (−) enantiomers, compound number CN: **9**) with an abundance of 25.37 ± 0.80%, *β*-pinene (CN: **5**) with 23.29 ± 0.15%, sabinene (CN: **4**) with 8.35 ± 0.10%, and oxygenated monoterpenes (OM, CF: C_10_H_18_O, MM: 154.14 Da), citronellal (CN: **21**) with 15.91 ± 0.76%, and 1,8-cineole (CN: **11**) with 6.19 ± 0.44%.

### 2.4. Enantiomeric Analysis

Using a gas chromatograph equipped with the MEGA-DEX DET Beta column of the enantioselective stationary phase, it was possible to separate four pairs of enantiomers in the EO from leaves of *Citrus* x *limonia*. Table 3 shows the retention time (RT), retention indices (RI), enantiomeric distribution (ED), and enantiomeric excess (e.e.) for each pair of compounds. For *α*-pinene and limonene one of the two enantiomers is found to be almost pure, with e.e. percentages of 98.28% and 96.75%, respectively.

Table 4 shows the results obtained using the MEGA-DEX DAC Beta enantioselective stationary phase column. This column was able to separate a pair of enantiomers.

### 2.5. Antibacterial Activity

The antibacterial activity of the EO from leaves of *Citrus* x *limonia* was evaluated against three Gram-positive cocci, a Gram-positive bacillus, and four Gram-negative bacilli using the microdilution broth method. Table 5 shows the minimum inhibitory concentration (MIC) values of the EO from leaves of *Citrus* x *limonia*, the positive control and the negative control. The EO from the leaves of *Citrus* x *limonia* indicated MIC values of 1000 µg/mL against Gram-positive cocci *Enterococcus faecalis* and Gram-positive bacillus *Lysteria monocytogenes*. All microorganisms show normal growth in the negative control.

### 2.6. Antifungal Activity

Table 6 shows the values for the minimum inhibitory concentrations (MICs) of the EO from leaves of *Citrus* x *limonia* against two fungi and yeasts, the positive control, and the negative control. The EO from leaves of *Citrus* x *limonia* had MIC values of 250 µg/mL against *Aspergillus niger*.

### 2.7. Antioxidant Activity

Ion radical 2,2′-azino-bis(3-ethylbenzothiazoline-6-sulfonic acid) (ABTS^•+^, method called ABTS), and radical 2,2-diphenyl-1-picrylhydrazyl (DPPH^•^, method called DPPH) were used to determine antioxidant activity of the EO from leaves of *Citrus* x *limonia*. Table 7 shows the scavenging capacity (SC_50_) of the EO, positive control (trolox) values expressed in µg/mL for ABTS and DPPH methods, as well as trolox equivalent antioxidant capacity (TEAC) values. In the DPPH assay, the SC_50_ was not reached at the maximum concentration tested of 10,000 µg/mL

### 2.8. Anticholinesterase Activity

**Figure 2:**
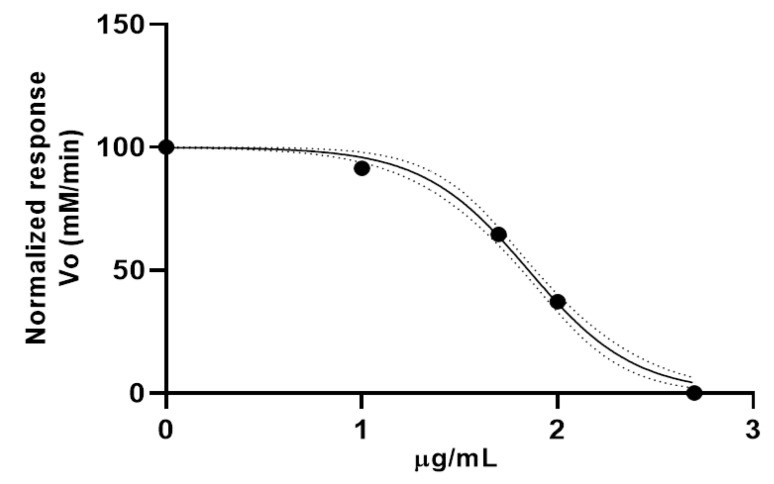
Anticholinesterase activity of essential oil from leaves of *Citrus* x *limonia*.

Anticholinesterase (anti-AChE) activity was determined using the spectrophotometric method. Figure 2 shows the rate of the reaction curve. The OE from leaves of *Citrus* x *limonia* exhibited a half-maximal inhibitory concentration (IC_50_) value of 71.02 ± 1.02 µg/mL. The positive control (donepezil) reported an IC_50_ value of 4.71 ± 0.51 µg/mL.

## 3. Discussion

The essential oil from leaves of *Citrus* x *limonia* was obtained by hydrodistillation with an extraction yield of 0.35 ± 0.02% (*v*/*w*) or 3.5 ± 0.2 mL/Kg; such yield is classified as low according the categorization system cited by Molares et al. in 2009 [21]. Essential oils are homogeneous mixtures of aromatic compounds, including terpenoid hydrocarbons, alcohols, ketones, ethers, phenolic compounds, monoterpenes, and sesquiterpenes. Their physical properties, such as color, density, refractive index, and specific rotation are influenced by the composition and relative proportions of these constituents. The apparent color of the essential oil was yellowish green, which is the characteristic color of essential oils derived from green leaves. The analytic measurements of the essential oil color are presented in Table 1. Color is a critical parameter in essential oils, which could serve as a standardization criterion when the oil is intended for use as an ingredient in food or cosmetic products, as it directly impacts consumer perception and product consistency [22]. The density of essential oils is generally lower than that of water due to their lipophilic nature, which facilitates their separation during hydrodistillation. The EO from leaves of *C.* x *limonia* shows a density of 0.9014 ± 0.0041 g/cm^3^.

The refractive index is an important quality parameter that reflects the chemical composition of the oil. It can be considered as the sum of the refractive indices of the individual components multiplied by the molar fractions [23]. For the EO from leaves of *C.* x *limonia*, the measured refractive index was 1.4885 ± 0.0019. This value aligns with the refractive indices of the major components such as limonene, which ranges from 1.34 to 1.56 [24], and *β*-pinene, reported at 1.4768 [25]. The specific rotation of the EO, a property that depends on the enantiomeric composition of its constituents, showed a value of +1.40 ± 0.07°. For comparison, (+)-limonene exhibits a specific rotation of +123.8° [26], while (−)-limonene shows −94.4° at 10% in ethanol [27]. The low specific rotation value of the EO suggests a mixture of enantiomers or the presence of other chiral compounds with lower optical activity. This property is particularly significant for evaluating the enantiomeric purity of essential oils, which can influence their biological activity, aroma, and potential therapeutic applications.

The *Citrus* genus is known for aromatic properties, with many species contributing EOs to various industries, such as pharmaceuticals, cosmetics, and food production [28,29]. The essential oils derived from *Citrus* species contain a variety of bioactive compounds, including monoterpenes such as limonene, *β*-pinene, and *γ*-terpinene [30,31,32]. The EO from leaves of *C.* x *limonia* was composed mainly of monoterpene hydrocarbons (65.67%) and oxygenated monoterpenes (28.70%). Five compounds, with an abundance of >5%, were identified as the major compounds, representing 79.11% of the total identified. These were limonene (25.37 ± 0.80%), *β*-pinene (23.29 ± 0.15%), sabinene (8.35 ± 0.10%), citronellal (15.91 ± 0.76%), and 1,8-cineol (6.19 ± 0.44%). Despite the variability of volatile compounds in essential oils, limonene is the characteristic major compound in *Citrus* species leaf essential oils, as can be observed when the chemical composition is compared with other studies. In 2016, Estevam et al. [33] reported the most abundant chemical components in the EO from fresh leaves of *C. limonia* Osbeck being limonene (40.0%), 1,8 cineol (13.4%), caryophyllene oxide (6.9%) and nerol (6.8%) compounds. In the same year, Moara et al. [34] reported the major compounds for the EO from leaves of *C. limonia* as limonene (29.9%), *β*-pinene (12.0%), sabinene (9.0%), citronellal (9.0%), and citronellol (5.8%). In 2022, El-Hawary et al. [35] presented the chemical composition of the EO from leaves of *C. medica* var. sarcodactylis, where the main components were limonene 32.11%, citral 28%, and nerol 27.8%. In 2024, Amala et al. [36] compared the chemical composition of five *Citrus* species leaf essential oils revealing that the main compound was limonene (15.76 ± 0.01% to 29.36 ± 0.46%), and that monoterpenoids (77.38 ± 0.19% to 98.05 ± 0.05%) were the predominant class of constituents. Additionally, the presence of non-volatile secondary metabolites such as coumarins and flavonoids was determined in the ethanolic extracts of *Citrus* x *limonia* [12].

The enantiomeric distribution of compounds in essential oils is critical for understanding their biological activity, organoleptic properties, and pharmacological effects [37,38]. The specific rotation of an essential oil is directly related to the proportion of chiral enantiomers it contains. Enantiomeric analysis of the EO from leaves of *C.* x *limonia* identified four pairs of enantiomers using the MEGA-DEX DET column: *α*-pinene, *β*-pinene, sabinene, and limonene. Additionally, two pairs of enantiomers were detected with the beta enantioselective column MEGA-DEX DAC: linalool and citronellal. In 2023, Cucinotta et al. [39] investigated the key terpenes in the essential oil of Sicilian lemon samples, and identified five pairs of enantiomers, including (−)-*β*-pinene (95.04–91.33%) and (+)-*β*-pinene (4.96–8.67%), (−)-limonene (1.65–2.02%) and (+)-limonene (98.35–87.89%), and (−)-linalool (48.00–75.66%) and (+)-linalool (52.00–24.34%). Similarly, Bhandari et al. [40], in 2024, reported the chiral distribution of the EO from leaves of *C. sinensis*, which exhibited 16 pairs of enantiomers. Among them were (−)-*β*-pinene (46.11%) and (+)-*β*-pinene (53.89%), (−)-sabinene (2.69%) and (+)-sabinene (97.31%), (−)-limonene (33.74%) and (+)-limonene (66.62%), and (−)-linalool (12.14%) and (+)-linalool (87.86%), while (+)-citronellal was found in its pure dextrorotatory form. The relationship between enantiomeric composition and the sensory and functional properties of essential oils highlights the critical role of chiral analysis in their characterization. For instance, (*S*)-(–)-limonene is responsible for the distinctive lemon aroma, while (*R*)-(+)-limonene, predominant in orange oil, contributes to its characteristic orange scent [27]. This highlights how the enantiomeric profile directly influences the sensory attributes of essential oils.

The essential oils from the *Citrus* genus are linked to antimicrobial, anti-inflammatory, antioxidant, anticarcinogenic, anthelmintic, insecticidal, and larvicidal properties, among others [30,31,32]. The EO from leaves of *C.* x *limonia* showed moderate antibacterial activity against *Enterococcus faecalis* and *Lysteria monocytogenes,* and strong antifungal activity against *Aspergillus niger* and *Candida albicans,* according to the scale presented in 2017 by Van Vuuren and Holl [41]. In 2016, Estevam et al. [33] reported the antimicrobial activity of EO from leaves of *C. limonia* Osbeck as MIC by broth dilution method against *Streptococcus mutants* (200 µg/mL), *S. mitis* (100 µg/mL), *S. sanguinis* (400 µg/mL), *S. sobrinus* (400 µg/mL) and *Bacteroides fragilis* (400 µg/mL). In another study of the EO of leaves of *C. medica,* it was reported to have good antimicrobial activity against *Bacillus subtilis*, *Bacillus cereus*, and *Staphylococcus aureus,* while showing lower activity against *Klebsiella pneumonia* [35]. In 2020, Chi et al. [42] reported the antimicrobial activity of three *Citrus* species leaf EOs using a diffusion assay. *C. grandis* showed strong antimicrobial activity against *S. aureus* (10.5 mg/mL), *Bacillus cereus* (5.25 mg/mL), and *Salmonella typhi* (5.25 mg/mL), while *C. sinensis* was active against *S. aureus* (5.25 mg/mL), *B. cereus* (10.5 mg/mL), and *S. typhi* (21 mg/mL). *C. aurantifolia* showed activity against *S. aureus* (21 mg/mL), *B. cereus* (10.5 mg/mL), and *S. typhi* (21 mg/mL). More recently, in 2014, Amala et al. [36] presented various degrees of antimicrobial activity in *Citrus* species leaf EO, reported based on the minimum inhibitory concentration using the disk diffusion method. The MIC value ranged between 7.02 ± 0.03 and 60 ± 0.32 mg/mL against *S. aureus*, between 3.62 ± 0.03 and 10.15 ± 0.02 mg/mL against *E. coli*, between 5.26 ± 0.02 and ≈ 27 mg/mL against *A. niger*, and from 3.62 ± 0.02 to 15.28 ± 0.04 mg/mL against *C. albicans*, with the EO from leaves of *C. aurantifolia* being the most active. The variety of antimicrobial activities reported for *C. species* demonstrates the dependence on the variety and kind of bacterium, in addition to its chemical composition, and interactions with the medium.

Regarding antioxidant activity, the EO from leaves of *C.* x *limonia* shows high SC_50_ values for ABTS (9115.15 ± 2.13 µg/mL) and DPPH (>10,000 µg/mL), which have low activity when compared to the control Trolox (ABTS 29.09 ± 1.05 µM and DPPH 35.54 ± 1.04 µM). These results showed lower antioxidant activity than the ones reported as SC_50_ for EOs of *C. aurantifolia* (1.21 mg/mL), *C. sinensis* (1.49 mg/mL), and *C. grandis* (2.18 mg/mL) [42]. On the other hand, in 2023, Petretto et al. [43] characterized the antioxidant activity of the EO leaves of *C. limon* by DPPH assay, and reported an SC_50_ value of 10.24 ± 2.8 mg/mL, which is similar to our results. The differences observed in antioxidant activity could be attributed to the chemical compositions seen among *Citrus* species. The antioxidant activity of the EO could be attributed to the synergistic antioxidant activity of the main compounds, which, in this case, are as follows: limonene, *β*-pinene, sabinene, citronellal and 1,8-cineole. The SC_50_ values for these components are different between references. In 2018, Shah et al. [44] presented, with regard to limonene, values of 384.73 μM (52.42 μg/mL) for DPPH and 603.23 μM (82.18 μg/mL) for ABTS, while in 2015, Sharopov et al. [45] reported a value of 6158.6 μg/mL for DPPH and 5893.2 μg/mL for ABTS, and for *β*-pinene, an SC_50_ of 3116.3 μg/mL for DPPH and 2245.0 μg/mL for ABTS.

The in vitro anticholinesterase activity of essential oils has been proven as an initial step in proposing applications against Alzheimer’s diseases [46]. Along the lines of other bioactivity assays, the response varies depending on the chemical composition. In 2019, Benny and Thomas [46] highlighted that most essential oils exhibit anticholinesterase properties, with antioxidant activity playing a protective role in preventing neuronal damage due to their ability to cross the blood–brain barrier. In this study, the anti-AChE activity, reported as IC_50_, was 71.02 ± 1.02 μg/mL, a value approximately five times higher than that of the control, donepezil (4.71 ± 0.51 μg/mL). Scientific reports of the anti-AChE activity of essential oils from *Citrus* species could be used for comparison: *C. aurantifolia* exhibited an IC_50_ of 19.57 ± 2.66 μg/mL while *C. x aurantium* showed an IC_50_ of 226.1 ± 7.41 μg/mL [47]. In 2011, Aazza et al. [48] reported an IC_50_ of 849.9 ± 11.5 μg/mL for *C. limon* peels EO, and Menichini et al. [49] presented an IC_50_ value of 171.3 μg/mL for *Citrus medica* L. cv Diamante peels EO. According to Santos et al. [50], the anti-AChE activity of the EO from leaves of *C.* x *limonia* has a moderate potency (20 < IC_50_ < 200 μg/mL).

## 4. Materials and Methods

### 4.1. Materials

Aliphatic hydrocarbons were purchased from ChemService (West Chester, PA, USA). Sabouraud dextrose broth, fluid thioglycollate medium, Mueller–Himton broth and Mueller–Hinton II broth were purchased from DIPCO (Quito, Ecuador). Helium was purchased from INDURA (Quito, Ecuador). 2,2-diphenyl-1-picrylhydryl (DPPH), 2,2′-azinobis-3-ethylbenzothiazoline-6-sulfonic acid (ABTS), 5,5′-dithiobis (2-nitrobenzoic acid) (DTNB), acetylcholinesterase (AChE), acetylthiocholine (AcSCh), butylated hydroxytoluene (BHT), dichloromethane (DCM), donepezil, dimethyl sulfoxide (DMSO), methanol (MeOH), magnesium chloride hexahydrate, phosphate-buffered saline (PBS), tris hydrochloride (Tris-HCl), sabouraud dextrose broth (SDB), sodium sulfate anhydrous and trolox were purchased from Sigma-Aldrich (San Luis, MO, USA). Reagents were of analytical grade and used directly without any additional purification.

### 4.2. Plant Material

The leaves of *Citrus* x *limonia* were collected on La Florida site, canton Palanda, province of Zamora Chinchipe. The site of collection is located at an altitude of 139 m.a.s.l. at 4°37′42.9″ south longitude, and 79°07′44.4″ west latitude. Once the plant material was collected, it was stored and transferred in airtight plastic containers to the university facilities. Botanist Nixon Cumbicus made the identification of the plant material. A voucher specimen with herbarium code HUTPL15058 was deposited at the Herbarium of Universidad Técnica Particular de Loja (HUTPL).

### 4.3. Postharvest Treatments

The plant material arrived four hours after being collected and was immediately subjected to postharvest treatment. This treatment involved the removal of foreign or deteriorated plant matter.

### 4.4. Moisture Determination

The determination of the humidity of the plant material was carried out according to Equation (1), using an analytical balance (Mettler AC 100, Mettler Toledo, Columbus, OH, USA) by the method of AOAC 930.04-1930 entitled Loss on dried (Moisture) in plants.

(1) $$Moisture\left(\%\right)=\frac{wi-wo}{wi}\times 100$$

where w is the “i”, initial, and “o”, after drying, weight sample .

### 4.5. Essential Oil Isolation

The EO was extracted through hydrodistillation using a Clevenger-type apparatus (80 L distiller, local construction). The process began by adding 16 L of water to the distiller, followed by the plant material. Extraction was conducted for 3 h, starting from the collection of the first drop of distillate. The resulting vapor, containing the EO and water, was condensed, and the OE was separated by decantation. In order to dry the oil, anhydrous sodium sulfate was used. The dried EO was then stored at 4 °C in sealed amber vials.

### 4.6. Determination of the Physical Properties of the Essential Oil

The following objective physical properties of the EO were determined: density, refractive index, and optical rotation, and the subjective physical property, color. The density of the EO was measured according to the AFNOR NF T 75-111 standard (equivalent to the ISO 279:1998 standard [51]) using an analytical balance (Mettler AC 100, Mettler Toledo, Columbus, OH, USA). Refractive index was determined following the AFNOR NF T 75-112 standard (similar to the ISO 280:1998 standard [52]) by means of a refractometer (model ABBE, BOECO, Hamburg, Germany). Optical rotation was measured according to the ISO 592:1998 standard [53]. For this purpose, an automatic polarimeter (Mrc-P810, MRC, Holon, Israel) was used. The subjective color of the EO was obtained online at the PINETOOL website https://pinetools.com/ (accessed on 24 October 2024) where a photograph of the EO with a white background had been uploaded. The analysis temperature for all properties was 20 °C.

### 4.7. Essential Oil Compounds Identification

#### Quantitative and Qualitative Analysis

The chemical composition of the EO from fruits of *C.* x *limonia* was determined using a gas chromatograph (Thermo Scientific, Trace 1310, Waltham, MA, USA) provided with Thermo Scientific Chromeleon 7.2 Chromatography Data System (CDS) software and NIST 17 and AMDIS 2.7 mass spectral libraries database. For quantitative analyses, the chromatograph was coupled to a flame ionization detector (GC-FID), whereas for qualitative analyses it was coupled to a mass spectrometer (quadrupole) detector (ISQ 7000, Thermo Scientific, Waltham, MA, USA) (GC-MS). In both cases, an automatic injector (AI 1310, Thermo Scientific, Waltham, MA, USA), and a nonpolar GC column (TR-5MS, Thermo Scientific, Waltham, MA, USA) with stationary phase 5%-phenyl-methylpolyxilosane, 0.25 µm of stationary layer thickness, 0.25 mm of diameter and 30 m of length, were used. A total of 1 µL of sample was injected at a concentration of 1/100 *v*/*v* (EO/DCM) with a split ratio of 1:50. Helium was used as a carrier gas, at 1 mL/min in constant flow mode and with an average velocity of 25 cm/s for quantitative analyses, and at 0.9 mL/min with an average velocity of 34 cm/s for qualitative analyses. The injector and detector temperatures in both analyses were 230 °C. The temperature ramp in the oven was similar in both analyses: 50 °C for 3 min, from 50 °C to 230 °C at 3 °C/min (60 min), and 230 °C for 3 min (total 66 min). The relative amounts of the compounds were calculated based on the GC peak area (FID response) without using a correction factor. In MS, a mass range of 40 at 350 *m*/*z*, electron multiplier 1600 eV, 70 eV, and a scan rate of 2 scan/s were used. The ion source temperature was set to 230 °C. Equation (2) [54] was used to determine the retention index (RI) of each compound. The identification of the compounds was carried out in two steps: first, by comparison of the mass spectra of the compounds in the sample with the spectra of the internal database, then by coincidence of the IR and mass spectra with published data [19,20].

(2) $$RI=100C+100\frac{\left(RTx-RTn\right)}{\left(RTN-RTn\right)}$$

where C is the carbon number of aliphatic hydrocarbons that elute before the compound of interest. RTx, RTn and RTN are the retention times of the compound of interest and the retention times of the aliphatic hydrocarbon that elutes before and after the compound of interest, respectively.

### 4.8. Enantioselective Analysis

The enantiomeric analysis of the EO from fruits of *C.* x *limonia* was performed on the same chromatograph, detector and injector as described for the qualitative analysis. To determine the enantiomeric distribution, two enantioselective GC columns of 30 m long, 0.25 m in internal diameter, and with 0.25 μm thick stationary phase were used; one was called MEGA-DEX DET-Beta (Mega, Legnano, MI, Italy), with diethyl tertbutylsilyl-beta-cyclodextrin stationary phase, and the other was called MEGA-DEX DAC-Beta (Mega, Legnano, MI, Italy), with diacetyl tertbutylsilyl-beta-cyclodextrin stationary phase. Split ration, gas carrier, flow velocity, injector and detector temperatures, as well as the temperature ramp and MS parameters, were the same as those described for the qualitative analysis. The elution order of the enantiomers of the compounds was determined based on the technical data of the column. The enantiomeric excess was calculated as the percentage of the major enantiomer minus the percentage of the minor enantiomer.

### 4.9. Antimicrobial Activity

#### 4.9.1. Determination of Antibacterial Activity

The antibacterial activity of the EO from fruits of *C.* x *limonia* was tested against three Gram-positive cocci: *Enterococcus faecalis* (ATCC 19433), *Enterococcus faecium* (ATCC 27270) and *Staphylococcus aureus* (ATCC 25923); a Gram-positive bacillus: *Lysteria monocytogenes* (ATTC 19115); and four Gram-negative bacilli: *Escherichia coli* O157:H7 (ATCC 43888), *Campylobacter jejuni* (ATCC 33560), *Pseudomonas aeruginosa* (ATCC 10145) and *Salmonella* enterica subs enterica serovar *Thypimurium* WDCM 00031, derived from (ATCC 14028). The tests were carried out as previously described by Valarezo et al., 2021 [55], using the microdilution broth method. In summary, the antibacterial assay was developed in a 96 microwell plate. Two-fold serial dilution was used to obtain a concentration of the EO ranging from 4000 to 15.62 µg/mL. For *Campylobacter jejuni*, decreasing concentrations of EO, from 4000 to 31.25 µg/mL, were used [56]. Ampicillin was used as a positive control for Gram-positive cocci, and ciprofloxacin for Gram-positive bacillus and Gram-negative bacilli. DMSO at 5% was used as a negative control. The maximum evaluated concentration was 4000 µg/mL MIC, and the lowest concentration of an antimicrobial which inhibited the growth of a microorganism after its incubation was used to report the activity values.

#### 4.9.2. Determination of Antifungal Activity

The antifungal activity of the EO from fruits of *C.* x *limonia* was tested against the fungus *Aspergillus niger* (ATCC 6275) and the yeast *Candida albicans* (ATTC 10231). The tests were carried out as previously described by Valarezo et al., 2021 [55] using the microdilution broth method. In summary, the antifungal assay was developed into a 96 microwell plate. Two-fold serial dilution was used to obtain concentration of the EO ranging from 4000 to 15.62 µg/mL MIC. The final concentration of spores was 5 × 104 spores/mL. The EO was dissolved in SDB with fungal inoculum to acquire the required concentrations. The maximum evaluated concentration was 4000 µg/mL; amphotericin B was used as a positive control, and DMSO as a negative control.

### 4.10. Evaluation of Antioxidant Capacity

#### 4.10.1. ABTS Radical Cation Scavenging Activity

The free radical scavenging activity of the EO from fruits of *C.* x *limonia* was determined using the ABTS method. This method, also known as trolox equivalent antioxidant capacity (TEAC) [57], uses the reagent ABTS to produce a ABTS radical cation (ABTS^•+^). The tests were carried out as previously described by Valarezo et al., 2021 [58], using the colorimetric method, and a UV spectrophotometer (Genesys 10S UV-Vis Spectrophotometer, Thermo Fisher Scientific, Waltham, MA, USA). In sum, the antiradical capacity of the EO was assessed against ABTS^•+^ by measuring the rate of reduction at a wavelength of 734 nm. Antiradical capacity was expressed as half scavenging capacity (SC_50_), calculated from the corresponding curve fitting. The maximum evaluated concentration was 2000 µg/mL. Trolox was used as a positive control, and MeOH as a negative control. Using the graph of % EO Inhibition vs. the Logarithm (Log10) of the concentration, the IC_50_ (concentration at which the % Inhibition is equal to 50%) of the essential oil was calculated. The TEAC value is obtained from the relationship between the IC_50_ of trolox in µM/mL, and the IC50 of the sample in g/mL.

#### 4.10.2. DPPH Radical Scavenging Activity

Free radical scavenging activity of the EO from fruits of *C.* x *limonia* was also determined with the DPPH method. In the DPPH method, the reagent DPPH was used to produce DPPH radical (DPPH^•^). The assays were carried out as previously described by Valarezo et al., 2021 [58]. The equipment, maximum concentration evaluated and negative and positive controls were the same as for ABTS method, with the difference being that the antiradical capacity of EO was assessed against DPPH^•^ by measuring the rate of reduction at a wavelength of 515 nm.

### 4.11. Determination of Anticholinesterase Activity

Anticholinesterase tests were carried out according to the principle described by Ellman et al. [59], as previously described by Valarezo et al. 2022 [56], using the microplate spectrophotometer (EPOCH 2, BioTek, Winooski, VT, USA). In sum, a mix of reactions containing buffer Tris 50 mM pH 8.0, acetylthiocholine, Ellman’s reagent DTNB and EO at different, decreasing concentrations were pre-incubated at 25 °C, for three minutes. Later, acetylcholinesterase was added to start reaction. The progression of the reaction was monitored at a wavelength of 412 nm. The IC_50_ (half inhibitory concentration) was extracted from the non-linear regression model (normalized response vs. log Inhibitor-variable slope). Donepezil was used as a positive control, and MeOH as a negative control.

### 4.12. Statistical Analysis

The data were collected on a Microsoft Excel sheet. The statistical software Minitab 17 (Version 17.1.0., Minitab LLC., State College, PA, USA) was used to calculate the measures of central tendency and standard deviation. Data from the evaluation of antioxidant capacity and anticholinesterase activity were analyzed by the GraphPad Prism, version 6.0 software (GraphPad Software Inc., San Diego, CA, USA). The procedures for essential oil isolation, physical properties evaluation, the evaluation of antioxidant capacity, and anticholinesterase activity evaluations were performed in triplicate. The identification of EO compounds, enantioselective analysis and antimicrobial activity were performed nine times.

## 5. Conclusions

This study provides the first comprehensive analysis of the chemical composition, enantiomeric distribution and biological activities of the essential oil from leaves of *Citrus* x *limonia*. A total of forty-three compounds were identified, with limonene as the predominant constituent. The essential oil exhibited strong antifungal activity, particularly against *Aspergillus niger* and *Candida albicans*, suggesting its potential as a natural antifungal agent. Additionally, it demonstrated moderate antibacterial activity against *Enterococcus faecalis* and *Lysteria monocytogenes*, indicating possible applications in controlling of these bacterial pathogens.

The moderate anticholinesterase activity observed suggests that *Citrus* x *limonia* essential oil may have potential in neuroprotective applications, particularly in the context of neurodegenerative diseases such as Alzheimer’s. The antioxidant properties further support its potential role in reducing oxidative stress-related damage, which is relevant in various clinical and pharmaceutical applications.

These findings contribute to a deeper understanding of Ecuadorian aromatic plants and their bioactive properties. Based on the biological properties of the main compounds, future studies of this essential oil could focus on activities such as anti-inflammatory, analgesic, neuroprotective, anxiolytic, repellent, insecticidal or antitumor. Additionally, research into the enantiomeric influence on bioactivity could provide further insights into its therapeutic potential.

## Figures and Tables

**Table 1:** Physical properties of the essential oil of *Citrus* x *limonia*.

	*Citrus* x *limonia* Leaf EO	
	Mean	SD
Density, ρ (g/cm^3^)	0.9014	0.0041
Refractive index, *n*^20^	1.4885	0.0019
Optical rotation, [*α*] (°)	+1.40	0.07
Color		
Subjective	yellowish green	
RGB color values	R:204, G:188, B:20	
CMYK color values	C:0, M:8, Y:90, K:20	
Hex Color Codes	#ccbc14	

**Table 2:** Chemical composition of the essential oil from leaves of *Citrus* x *limonia*.

CN	Compound	RIC	RIR	%	SD	Type	CF	MM (Da)
1	Thujene <*α*->	924	924	0.14	0.01	MH	C_10_H_16_	136.13
2	Pinene <*α*->	932	932	1.86	0.20	MH	C_10_H_16_	136.13
3	Camphene	946	946	0.13	0.00	MH	C_10_H_16_	136.13
4	Sabinene	970	969	8.35	0.10	MH	C_10_H_16_	136.13
5	Pinene <*β*->	975	974	23.29	0.15	MH	C_10_H_16_	136.13
6	Myrcene	988	988	1.09	0.07	MH	C_10_H_16_	136.13
7	Octanal <*n*->	998	998	0.12	0.00	OC	C_8_H_16_O	128.12
8	Terpinene <*α*->	1015	1014	0.11	0.01	MH	C_10_H_16_	136.13
9	Limonene	1024	1024	25.37	0.80	MH	C_10_H_16_	136.13
10	Phellandrene <*β*->	1026	1025	0.73	0.04	MH	C_10_H_16_	136.13
11	Cineole <1,8->	1028	1026	6.19	0.44	OM	C_10_H_18_O	154.14
12	Ocimene <(*Z*)-*β*->	1030	1032	0.60	0.11	MH	C_10_H_16_	136.13
13	Ocimene <(*E*)-*β*->	1043	1044	3.12	0.32	MH	C_10_H_16_	136.13
14	Terpinene <*γ*->	1053	1054	0.72	0.07	MH	C_10_H_16_	136.13
15	Sabinene hydrate <*cis*-> (IPP vs. OH)	1067	1065	0.20	0.00	OM	C_10_H_18_O	154.14
16	Mentha-2,4(8)-diene <*ρ*->	1086	1085	0.10	0.01	MH	C_10_H_16_	136.13
17	Linalool	1096	1095	1.25	0.03	OM	C_10_H_18_O	154.14
18	Nonanal <*n*->	1102	1100	0.29	0.00	OC	C_9_H_18_O	142.14
19	Menthatriene <1,3,8-*ρ*->	1109	1108	0.06	0.01	MH	C_10_H_14_	134.11
20	Sabina ketone <*dehydro*->	1117	1117	0.39	0.03	OC	C_9_H_12_O	136.09
21	Citronellal	1149	1148	15.91	0.76	OM	C_10_H_18_O	154.14
22	Isopulegol <*neoiso*->	1169	1167	0.10	0.01	OM	C_10_H_18_O	154.14
23	Isocitral <(*Z*)->	1161	1160	0.06	0.00	OM	C_10_H_16_O	152.12
24	Terpinen-4-ol	1176	1174	0.39	0.03	OM	C_10_H_18_O	154.14
25	Terpineol <*α*->	1188	1186	1.70	0.03	OM	C_10_H_18_O	154.14
26	Decanal <*n*->	1202	1201	0.21	0.01	OM	C_10_H_20_O	156.15
27	Citronellol	1224	1223	0.97	0.02	OM	C_10_H_20_O	156.15
28	Neral	1237	1235	0.75	0.02	OM	C_10_H_16_O	152.12
29	Geranial	1265	1264	0.96	0.01	OM	C_10_H_16_O	152.12
30	Undecanal	1307	1305	0.19	0.03	OC	C_11_H_22_O	170.17
31	Guaiacol <*ρ-vinyl*->	1311	1309	0.06	0.01	OC	C_9_H_10_O_2_	150.07
32	Citronellyl acetate	1351	1350	0.83	0.15	OC	C_12_H_22_O_2_	198.16
33	Neryl acetate	1360	1359	0.21	0.02	OC	C_12_H_20_O_2_	196.15
34	Geranyl acetate	1380	1379	0.17	0.01	OC	C_12_H_20_O_2_	196.15
35	Caryophyllene <(*E*)->	1418	1417	0.84	0.28	SH	C_15_H_24_	204.19
36	Bergamotene <*α*-*trans*->	1433	1432	tr	-	SH	C_15_H_24_	204.19
37	Humulene <*α*->	1453	1452	0.10	0.03	SH	C_15_H_24_	204.19
38	Germacrene D	1482	1480	0.11	0.01	SH	C_15_H_24_	204.19
39	Farnesene <(*E*,*E*)-*α*->	1506	1505	0.98	0.04	SH	C_15_H_24_	204.19
40	Germacrene A	1510	1508	0.10	0.01	SH	C_15_H_24_	204.19
41	Cadinene <*δ*->	1523	1522	0.07	0.00	SH	C_15_H_24_	204.19
42	Nerolidol <(*E*)->	1563	1561	0.06	0.01	OS	C_15_H_26_O	222.20
43	Apofarnesol <(*Z*)-*dihydro*->	1570	1571	0.23	0.20	OC	C_14_H_26_O	194.20
	Monoterpene hydrocarbons (MH)			65.67				
	Oxygenated monoterpenes (OM)			28.70				
	Sesquiterpene hydrocarbons (SH)			2.20				
	Oxygenated sesquiterpene (OS)			0.06				
	Other compounds (OC)			2.49				
	Total identified			99.13				

CN: compound number, assigned according to their elution order; RIC: calculated retention indices; RIR: retention indices based on literature; %: relative abundance; CF: chemical formula; MM: monoisotopic mass; SD: standard deviation; tr: traces.

**Table 3:** Chiral compounds present in the essential oil from leaves of *Citrus* x *limonia*, determined using the MEGA-DEX DET Beta column.

RT	RI	Enantiomers	ED (%)	e.e (%)
3.81	938	(+)-(1*R*,5*R*), (+)-(1*S*,5*S*), *α*-Pinene	0.86	98.28
3.89	940		99.14	
5.15	992	(+)-(1*R*,5*R*)-*β*-Pinene	41.28	17.44
5.17	993	(−)-(1*S*,5*S*)-*β*-Pinene	58.72	
5.35	1000	(+)-(1*R*,5*R*), (+)-(1*S*,5*S*), Sabinene	5.62	88.76
5.41	1002		94.38	
7.41	1052	(−)-(4*S*), (+)-(4*R*), Limonene	1.62	96.75
7.48	1054		98.38	

RT: retention time; RI: retention indices; ED: enantiomeric distribution; e.e.: enantiomeric excess.

**Table 4:** Chiral compounds in the essential oil from leaves of *Citrus* x *limonia,* determined using the MEGA-DEX DAC Beta column.

RT	RI	Enantiomers	ED (%)	e.e (%)
30.35	1199	(−)-(3*R*), (+)-(3*S*), Linalool	76.37	52.74
30.56	1200		23.63	
33.27	1231	(−)-(3*S*), (+)-(3*R*), Citronellal	5.523	88.95
33.48	1235		94.477	

RT: retention time; RI: retention indices; ED: enantiomeric distribution; e.e.: enantiomeric excess.

**Table 5:** Antibacterial activity of essential oil from leaves of *Citrus* x *limonia*.

Microorganism	*Citrus* x *limonia* Leaf EO	Positive Control *	Negative Control
	MIC (µg/mL)		
Gram-positive cocci			
*Enterococcus faecalis* (ATCC 19433)	1000	0.78	+
*Enterococcus faecium* (ATCC 27270)	4000	0.39	+
*Staphylococcus aureus* (ATCC 25923)	>4000	0.39	+
Gram-positive bacillus			
*Lysteria monocytogenes* (ATTC 19115)	1000	1.56	+
Gram-negative bacilli			
*Escherichia coli* O157:H7 (ATCC 43888)	4000	1.56	+
*Campylobacter jejuni* (ATCC 33560)	2000	1.56	
*Pseudomonas aeruginosa* (ATCC 10145)	>4000	0.39	+
*Salmonella enterica* subs enterica serovar Thypimurium WDCM 00031, derived (ATCC 14028)	>4000	0.39	+

* ampicillin for Gram-positive cocci and ciprofloxacin for Gram-positive bacillus and Gram-negative bacilli; +: normal growth.

**Table 6:** Antimicrobial activity of essential oil from leaves of *Citrus* x *limonia*.

Microorganism	*Citrus* x *limonia* leaf EO	Positive Control *	Negative Control
	MIC (µg/mL)		
Fungi			
*Aspergillus niger* (ATCC 6275)	250	0.098	+
Yeasts			
*Candida albicans* (ATTC 10231)	500	0.098	+

* Amphotericin B; MIC: minimum inhibitory concentration; +: normal growth.

**Table 7:** Antioxidant activity of essential oil from leaves of *Citrus* x *limonia*.

Sample	ABTS	DPPH	TEAC
	SC_50_ ± SD (µg/mL) *		Mean ± SD µM TE/g
*Citrus* x *limonia* leaf EO	9115.15 ± 2.13	>10,000	4.69 ± 0.04
Trolox	29.09 ± 1.05	35.54 ± 1.04	

ABTS: 2,2′-azinobis(3-ethylbenzothiazoline-6-sulfonic acid); DPPH: 2,2-diphenyl-1-picrylhydrazyl; TEAC: Trolox equivalent antioxidant capacity; TE: trolox equivalent; * µM for trolox; SD: standard deviation.

## Data Availability

Data are available from the authors upon reasonable request.
